# Non-destructive characterization of adult zebrafish models using Jones matrix optical coherence tomography

**DOI:** 10.1364/BOE.455876

**Published:** 2022-03-17

**Authors:** Antonia Lichtenegger, Pradipta Mukherjee, Lida Zhu, Rion Morishita, Kiriko Tomita, Daisuke Oida, Konrad Leskovar, Ibrahim Abd El-Sadek, Shuichi Makita, Stefanie Kirchberger, Martin Distel, Bernhard Baumann, Yoshiaki Yasuno

**Affiliations:** 1 Center for Medical Physics and Biomedical Engineering, Medical University of Vienna, Austria; 2 Computational Optics Group, Institute of Applied Physics, University of Tsukuba, Japan; 3 Department of Physics, Faculty of Science, Damietta University, Egypt; 4 St. Anna Children’s Cancer Research Institute (CCRI), Austria

## Abstract

The zebrafish is a valuable vertebrate animal model in pre-clinical cancer research. A Jones matrix optical coherence tomography (JM-OCT) prototype operating at 1310 nm and an intensity-based spectral-domain OCT setup at 840 nm were utilized to investigate adult wildtype and a tumor-developing zebrafish model. Various anatomical features were characterized based on their inherent scattering and polarization signature. A motorized translation stage in combination with the JM-OCT prototype enabled large field-of-view imaging to investigate adult zebrafish in a non-destructive way. The diseased animals exhibited tumor-related abnormalities in the brain and near the eye region. The scatter intensity, the attenuation coefficients and local polarization parameters such as the birefringence and the degree of polarization uniformity were analyzed to quantify differences in tumor versus control regions. The proof-of-concept study in a limited number of animals revealed a significant decrease in birefringence in tumors found in the brain and near the eye compared to control regions. The presented work showed the potential of OCT and JM-OCT as non-destructive, high-resolution, and real-time imaging modalities for pre-clinical research based on zebrafish.

## 1. Introduction

The zebrafish is a valuable animal model in pre-clinical research, especially in the field of cancer investigations [1,2]. In particular, the advantages of using zebrafish over mammalian models such as mice are their high fecundity; the female zebrafish can lay up to 200-300 eggs per week, the external fertilization and development and their transparency at embryonic and larval stages; which allows for live imaging in an intact vertebrate organism. Furthermore, genetic manipulation techniques are well established, and the zebrafish has a high genetic and organ system similarity to humans [2,3]. For cancer research, the possibility to perform forward and reverse genetic analyses in zebrafish models enables novel insights into the molecular genetics of tumors and to develop zebrafish models of different types of cancers [2]. Zebrafish models can be utilized to monitor the progression of the tumor growth *in vivo* and for drug discovery screens [4]. One key aspect is, that tumors formed in zebrafish histologically and genetically resemble human cancer types. Foremost, in comparison to the usage of other animal models such as mice, zebrafish investigations are cost and time effective [5,6].

Various imaging techniques have been utilized to investigate adult zebrafish models *in vivo* including microscopic imaging techniques [7], magnetic resonance imaging (MRI) [8], micro computed tomography (CT) [9] and other tomography based imaging approaches [10–12].

Microscopic techniques such as fluorescence microscopy offer high resolution and are compatible with markers which enable tissue specific contrast [13].

However, the generation of three-dimensional (3D) data sets is time consuming, large amounts of data must be acquired. In addition, the depth imaging range of standard microscopy is limited to a couple of hundred micrometers [14]. To overcome the image depth limitation in adult zebrafish, transparent models have been introduced through genetic modifications, leading however to additional working steps and more complicated breeding schemes [15,16].

In comparison to the microscopic techniques, micro MRI or CT both offer the possibility to investigate the whole zebrafish *in vivo*. In addition, paramagnetic markers can be used with MRI to generate tissue specific contrast [8,17]. However, micro-MRI and CT have several limitations such as the requirement of exogenous markers, the complexity of these imaging setups, the high cost involved, and invasiveness caused by the radiation or high magnetic fields [9,18].

Over the last decades multiple tomography-based imaging approaches have been introduced. For example, optical projection tomography and photoacoustic imaging have been recently proven to be useful to investigate adult zebrafish. However, the resolution of such techniques is limited to ten to hundred micrometers [11,19,20]. To investigate the whole adult zebrafish, an imaging modality which can offer 3D, high-imaging-depth, high-resolution, non-destructive, and tissue specific contrast without markers would be beneficial.

Optical coherence tomography (OCT) is a non-invasive optical imaging technique based on low-coherence interferometry. The image contrast is generated by the back scattering and reflection of light by the tissue morphology. OCT provides a label-free and three-dimensional method to investigate the tissue structure. Moreover, OCT is a real-time imaging modality which allows to acquire 3D data sets in a few seconds [21]. The axial or depth resolution of an OCT setup depends on the used light source and typically ranges from 1-15 micrometers [21–23]. In OCT based microscopy a large numerical aperture (NA) objective lens is utilized to achieve a high transverse resolution [24]. The possible imaging range in depth strongly depends on the used light source. In the near infrared wavelength region, depth ranges of couple of millimeters can be achieved [25]. The high resolution, high depth-imaging range, volumetric, label-free, and real time imaging capability of OCT make the technique a great candidate for biological studies. As such, OCT has been utilized in a wide range of applications, covering *ex vivo* tissue samples [26,27], *in vitro* spheroid [28,29], and *in vivo* animal model investigations [30,31].

Polarization sensitive OCT (PS-OCT) is a functional extension of conventional intensity-based OCT. Using PS-OCT, additional tissue specific contrast can be gained by analyzing the polarization states of the back-scattered light [32,33]. Two essential polarization effects which are often analyzed are the birefringence and the depolarization. In a birefringent material different polarization states experience different speeds of light. This effect can for example be observed in fibrous tissue structures [34,35].

The random change of the incident polarization state at spatially adjacent sample locations is called depolarization or also polarization scrambling. In PS-OCT the randomization of polarization states among neighboring speckles is thereby examined using the so-called degree of polarization uniformity (DOPU). DOPU has for example been shown to be a specific marker to detect melanin in the retinal pigment epithelium [36]. PS-OCT has widely been used to investigate polarization properties of various tissue types such as the retina [37], the skin [38], muscles, tendons and bones [39–41].

Furthermore, OCT and PS-OCT have been employed to investigate zebrafish. The existing studies can be divided into investigations performed in early development stages such as larvae [42–44] and adult zebrafish [45–49]. A big part of the studies performed in adult zebrafish models was either focused on the brain or on the retina. In general, most studies so far were conducted using intensity-based OCT setups. In 2015, Rossignoli *et al.* presented the first PS-OCT images of adult zebrafish using a light source centered at 1550 nm [50]. More recently, Yang *et al.* introduced a PS-OCT setup at a central wavelength of 840 nm to study various muscle groups and the skin in adult zebrafish [51,52].

The PS-OCT setups utilized so far measured the birefringent polarization property as cumulative phase retardation in addition to the backscattering intensity [50,52]. The cumulative phase retardation can be affected by superior tissue structures and can therefore falsify birefringence values in deeper tissue regions. Recently, an extended version of PS-OCT, Jones-matrix OCT (JM-OCT), has overcome this limitation via quantifying the local birefringence of the tissue by using a local Jones matrix analysis [32,33].

In this article we present the investigation of adult zebrafish using two different OCT setups, a custom-built JM-OCT prototype operating at 1310 nm and a spectral domain (SD) OCT setup at 840 nm. Utilizing the SD-OCT setup anatomical structures can be investigated by a higher axial and transversal resolution compared to the JM-OCT prototype. The advantages of utilizing JM-OCT in comparison to conventional intensity-based OCT to image anatomical structures found in the adult zebrafish were investigated. Furthermore, a motorized translation stage in combination with the JM-OCT prototype enabled large field-of-view imaging to non-destructively investigate a large part of the adult zebrafish body in less than a minute. Wildtype animals and tumor bearing zebrafish models were examined. The scatter intensity, the attenuation coefficient and local polarization parameters namely the birefringence and the DOPU were quantitatively analyzed to investigate differences found in tumor versus control specimens.

## 2. Methods

### 2.1 Jones matrix OCT setup

A custom-built JM-OCT setup was utilized to investigate adult zebrafish, details of the setup can be found elsewhere [38]. The JM-OCT prototype was based on a passive-polarization-delay-based PS-OCT scheme. A swept source laser with a central wavelength of 1310 nm (AXA10823-8, Axsun Technologies, MA) was used for imaging.

The A-scan rate was 50 kHz and a system sensitivity of 104 dB was measured with a probe beam power of 11 mW. The axial resolution in tissue was 14 μ m with a depth pixel separation of 7.24 μ m. The depth range in air was 2.9 mm. For imaging two alternative scan lenses were utilized, featuring effective numerical apertures (NA) and focal lengths of 0.048 and 36 mm (LSM03 scanning lens, Thorlabs, denoted as low-resolution scanning lens) and 0.097 and 18 mm (LSM02 scanning lens, Thorlabs, denoted as high-resolution scanning lens), respectively. The respective depth of focus values in air for the LSM03 and the LSM02 scanning lens are 0.39 mm and 0.09 mm respectively. The lateral resolutions for these scanning lenses were 18.1 μ m and 8.9 μ m, respectively. Data sets comprised 512 × 128 or 512 × 512 pixels. The imaged field-of-view (FoV) varied from 1 mm × 1 mm up to 6 mm × 6 mm.

### 2.2 840-nm SD-OCT setup

The fiber-based SD-OCT setup utilized an infrared light source centered at 840 nm (SLD, IW-M- D840-HP-I-CUS, Superlum) with a bandwidth of 100 nm, details can be found elsewhere [53]. The axial resolution in air was measured to be 5.3 μ m with an depth-pixel separation of 2.5 μ m. For imaging a scanning lens (LSM02-BB scanning lens, Thorlabs) with an effective focal length of 18 mm, providing a lateral resolution of 4.9 μ m and a depth of field of 30 μ m, was utilized. The interference fringes were collected by a spectrometer (CS800-850/140-250-OC2K-CL, Wasatch Photonics), running at an A-scan rate of 50 kHz.

The sensitivity of the setup was measured to be 104 dB and the roll-off in air was 3.8 dB per mm. The power at the sample was 6.3 mW. The depth range in air was 2.5 mm. The volumetric OCT data comprised 512 × 512 × 2048 pixels and the FoV varied from 1 mm × 1 mm up to 3 mm × 3 mm.

### 2.3 Data processing

**Fig. 1.**
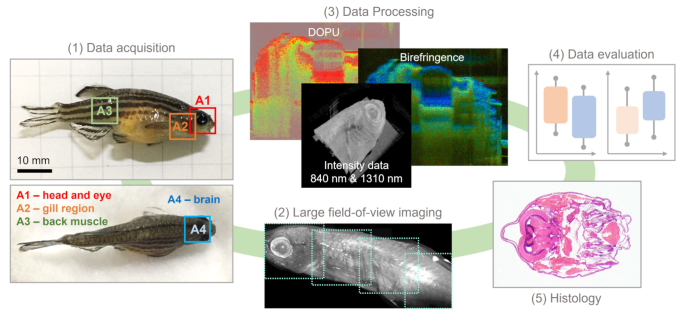
The data acquisition and processing steps. (1) The four indicated locations (A1-A4) were imaged to investigate different anatomical features of the zebrafish. (2) Large field-of-view images were acquired using a motorized translation stage. (3) Data post-processing was performed to retrieve images based on the local scattering and polarization properties of the samples. (4) Quantitative data analysis was performed to distinguish abnormal and control tissue areas. (5) After OCT imaging histological analysis was conducted.

An overview over the data processing steps involved is shown in Fig. 1. For the JM-OCT system four contrasts were investigated, the OCT scattering intensity, the attenuation coefficient, the local birefringence, and the degree of polarization uniformity (DOPU). To generate the scatter intensity-based data (in the manuscript referred to as the intensity data), the absolute-squared intensities of the four Jones matrix entries, corresponding to the four polarization channels, were averaged.

The attenuation coefficients were calculated from these intensity data by using the depth-resolved method demonstrated by Vermeer *et al.* [54]. The attenuation values are a quantitative representation of the absorption and scattering properties of an investigated sample.

The depth-resolved birefringence data, also known as the local retardation, were obtained by a local Jones matrix analysis in combination with a maximum *a posteriori* birefringence estimator [34,55]. To visualize the data, a pseudo-color birefringence tomogram is created by combining the scattering intensity, birefringence, and reliability of the birefringence estimation. In the images shown, the birefringence is expressed by color hue if it is reliable, otherwise the pixel is a shade of gray.

Finally, the DOPU values were calculated [56]. For visualization a composite image of the scatter intensity and DOPU data was generated, leading to mostly grey pixel values in the background region.

For the 840-nm SD-OCT data, standard OCT post-processing steps were performed, which included k -linear resampling, spectral shaping by multiplying with a Gaussian window, numerical dispersion compensation, average-spectrum subtraction and Fourier transformation to retrieve the OCT intensity images.

For both JM-OCT and 840-nm SD-OCT, Fiji [57] was utilized for data visualization. The depth ranges for the averaged *en face* projections are indicated by yellow triangles in the corresponding B-scan images throughout the manuscript.

To quantify the four parameters, measured with the JM-OCT prototype, in tumor versus control tissue areas, the following steps were performed. ITK-Snap [58], an open-source software tool, was used to manually segment the tumor and control regions intensity B-scan wise as referring to the histology as a ground truth. The resulting binary masks were applied to the original volumetric PS-OCT data (intensity, attenuation coefficient, birefringence and DOPU) and used to analyze the distribution of these values. All statistical evaluation was performed in MATLAB (MATLAB, R2021a, MathWorks). Box-whisker plots were created, showing the median values (red line), the 25%−75% percentiles (box), the maximum and minimum values (horizontal bars) and outliers (red crosses) of the data. Mann–Whitney U tests were performed to test for the equality of the distributions of the data using a significance level of p < 0.05. Additionally, a Levene’s test was used to evaluate the equality of the variances using a significance level of p < 0.05.

### 2.4 Large field-of-view imaging

Large FoV images were acquired with the JM-OCT prototype in combination with a motorized translation stage (MLS203-1, Thorlabs) moving in x and y direction. The stage was integrated into the sample arm and was able to scan large field-of-views up to several square centimeter. The maximal range of the stage was 11.0 cm × 7.5 cm. A custom-made LabView (LabView 2015, Version 15.0, 64-bit, National Instruments) program controlled the movement of the translation stage [59]. The OCT data were acquired with an overlap, in the lateral direction, of 5% and after post-processing the volumes were stitched together using the Pairwise stitching plugin of Fiji [60].

### 2.5 Zebrafish models

Zebrafish were maintained at standard conditions under the license GZ:759147-2017-13 and GZ:565304-2014-6 [61]. Tumors were introduced by injecting $HRAS^{G12V}$ oncogene containing plasmids (UAS: GFPHRASG12V or H2BCFP:UAS:HRASG12V at 10ng/ μ l) into fertilized eggs of transgenic Et(SP8b:KalTA4-UAS:mcherry) zebrafish [62]. By this means, mosaic expression of $HRAS^{G12V}$ was directed to the central nervous system. In total three adult control wildtype (AB) and five tumor-bearing SP8bRas zebrafish were investigated. One of those five fish exhibited tumor growth next to the eye as well as in the brain. Tumor fish were sacrificed between 3 to 12 months of age. The fish were fixated in 4% paraformaldehyde.

For imaging the fish were placed on a petri-dish and ultrasound gel was applied for index matching to reduce strong surface reflections. The anatomical features of four areas, namely the eye (A1), the gills (A2), the muscles and the brain (A4), indicated in Fig. 1 were investigated. For the regions A1 - A3 the fish was imaged lying on the side. For the region A4 the fish was imaged from the top. Following OCT measurements, histological workup was performed. Hematoxylin and eosin (H&E) stained micrographs were acquired with a standard transmission microscope (Olympus, SZX16) using 4X and 10X magnification objective lenses.

## 3. Results

### 3.1 Imaging of control zebrafish

**Fig. 2.**
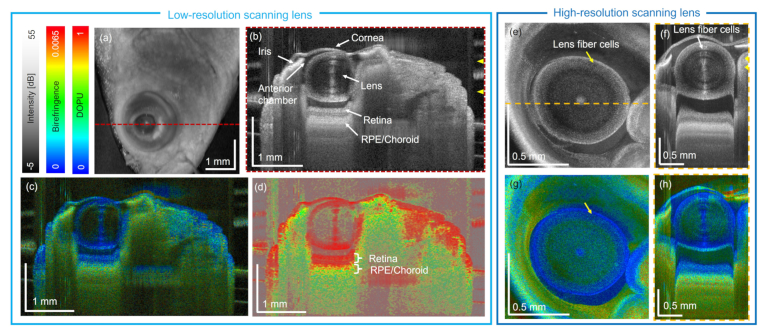
Zebrafish eye investigation using 1310-nm JM-OCT. (a) Intensity en-face projection in the lens region. (b) - (d) Central intensity, birefringence and DOPU tomograms. (e) Intensity en-face projection in the lens region imaged with the high-resolution scanning lens. (f) Central intensity B-scan image (RPE - retinal pigment epithelium). (g) - (h) The birefringence en-face and B-scan image. Dashed lines indicate the B-scan locations. Notice that the image data were not corrected for refraction caused by the eye media.

**Fig. 3.**
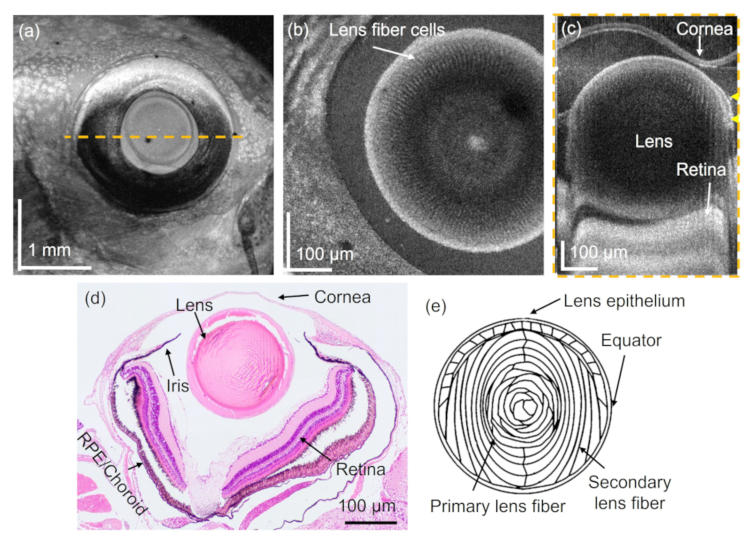
Zebrafish eye investigation using 840-nm SD-OCT. (a) Intensity en-face projection. The yellow dashed line indicates the B-scan location for (c). (b) En-face projection in the lens region. (c) Corresponding B-scan image to (a). (d) H&E-stained micrograph showing the anatomical features of the zebrafish eye in a transverse plane. (e) Graphical representation of the fiber cell orientation in an adult zebrafish lens. The graphic was drawn based on [63].

The eye region of the control zebrafish was investigated using the 1310-nm JM-OCT prototype. The results in Fig. 2(a) - (d) were obtained by using the low-resolution and Fig. 2(e) - (h) with the high-resolution scanning lenses, respectively. Figure 2(a) shows the intensity en-face projection and Fig. 2(b) - (d) the corresponding B-scan intensity, birefringence and DOPU images (indicated by the red dashed line), respectively. The anatomical features of the eye, such as the lens, the cornea, the iris, the anterior chamber, the retina and the retinal pigment epithelium (RPE) choroid complex can be identified, see Fig. 2(b) and (f), respectively. These structures can be compared to an H&E-stained histological micrograph shown in Fig. 3(d). Due to the postmortem imaging process the cornea got deformed. Further, an axial shift of the retinal structures due to the refractive properties of the lens and the aqueous can be observed, see Fig. 2(b) and (f), respectively.

The birefringence and DOPU images are presented in Fig. 2(c) - (d), respectively. The polarization properties of the fisheye can be identified, and it can be observed that the retina exhibits low birefringence and a high degree of polarization uniformity in comparison to RPE/choroid region. Furthermore, multiple scattering and depolarization trails can be observed beneath the iris and RPE/choroid complex. In the images acquired with the high-resolution scanning lens, Fig. 2(e) - (h), additionally fiber cells inside the lens can be identified.

The eye region of the wildtype zebrafish was further investigated using the 840-nm SD-OCT setup. An intensity en-face projection of a 3 mm × 3 mm FoV is shown in Fig. 3(a). A smaller FoV (1 mm × 1 mm) en-face projection is shown in Fig. 3(b) and the corresponding B-scan image in Fig. 3(c), respectively. The lens fiber cells can be observed as highly scattering regions in the en-face projection in Fig. 3(b). The orientation of the lens fiber cells can be correlated to the graphical representation of these structure in Fig. 3(e). The anatomical structures such as the cornea, the lens, and the retina in the corresponding B-scan image, see Fig. 3(c) can be correlated to the ground truth obtained by the H&E-stained histology micrograph, see Fig. 3(d).

**Fig. 4.**
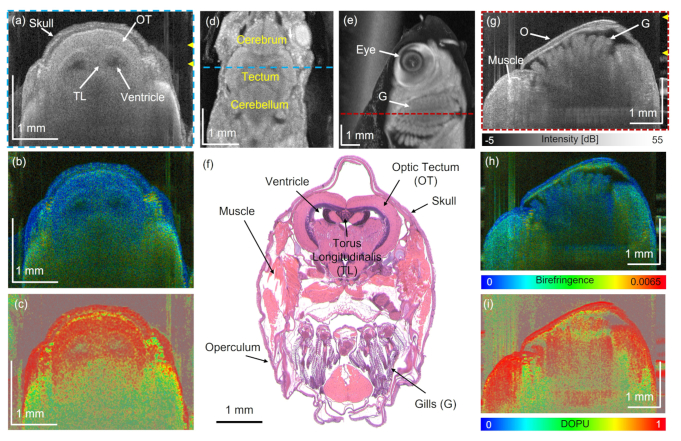
Brain and gill region in the wildtype zebrafish imaged with the JM-OCT prototype. Intensity (a), birefringence (b) and DOPU (c) B-scan images in the brain (OT- optic tectum, TL - torus longitudinalis). (d) Corresponding en-face intensity projection in the brain region. (e) En-face intensity projection in the gill region (G - gills). (f) H&E-stained micrograph. Corresponding intensity (g), birefringence (h) and DOPU (i) B-scan images of (b) (O - operculum, G - gills). Dashed lines indicate the B-scan locations.

Next, the brain and the gill region in the wildtype zebrafish were investigated. First the JM-OCT setup was utilized to investigate those anatomical locations. Figure 4(d) shows an en-face projection in the brain region and Fig. 4(a) and (b) the corresponding intensity and birefringence tomograms, respectively. Various anatomical features of the brain including the optic tectum (OT), the torus longitudinalis (TL) and the ventricles can be identified in the intensity-based B-scans and en-face projections (Fig. 4(a) and (d)) due to their different scattering characteristics.

**Fig. 5.**
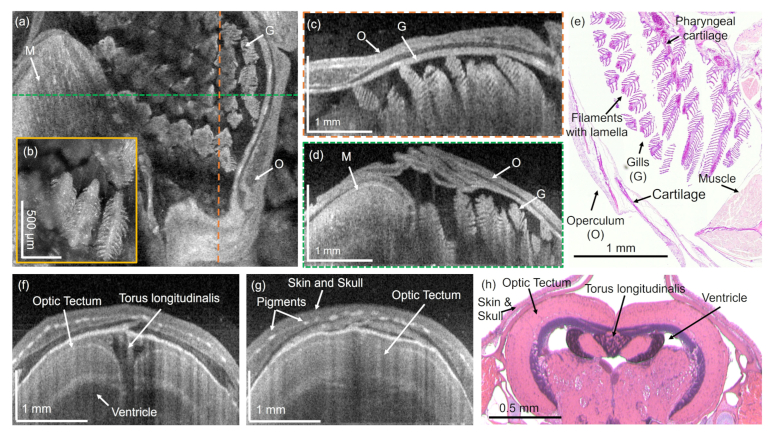
Zebrafish gill and brain region imaged with 840-nm SD-OCT. (a) En-face projection in the gill region and (b) a zoom-in into the gill structures. (c) Sagittal intensity-based tomogram in the gill region. (d) B-scan image showing the transverse section of the gills (O – operculum, G – gills, M - muscles). (e) H&E-stained histology image of the gill region. (f) and (g) tomograms of the zebrafish brain. (h) Representative H&E-stained histology micrograph. Dashed lines indicate the tomogram locations.

The ventricles appeared as hypo-scattering regions while myelinated areas such as the TL exhibit higher scattering. In the corresponding birefringence tomogram, Fig. 4(b), fiber-rich areas in the brain exhibited higher birefringence. The H&E-stained histology image confirmed these findings, see Fig. 4(f). Figure 4(e) shows an en-face projection in the head and the gill region. The gills can be identified by their characteristic morphology, which can be compared to the appearance in the histology image shown in Fig. 4(f). Figure 4(g) and (h) show the corresponding intensity and birefringence B-scan images in the gill region. In Fig. 4(h) and (i) the operculum, the bony plate covering the sensitive gills, can be identified by a polarization preserving and highly birefringent layer. The observed layers structure of the operculum can be compared to the histology micrograph in Fig. 5(e). Further, the gill filaments exhibit a lower birefringence in comparison to adjacent muscle region.

Hereafter, the gill and the brain region were also imaged utilizing the 840-nm SD-OCT system. Figure 5(a) shows the intensity en-face projection in a 3 mm × 3 mm FoV and Fig. 5(b) a zoom-in into the gill structures. Utilizing the higher resolution of the SD-OCT setup the fine branches and respiratory lamellae of the gills can be visualized and compared to an H&E-stained micrograph, see Fig. 5(e). Figure 5(c) and (d) show the intensity-based tomograms in selected transverse (orange) and sagittal (green) planes. In both B-scan images the fine details of the branches, the filaments covering the lamellae of the gills can be investigated. Furthermore, the brain region was examined with the SD-OCT setup. Two selected B-scan images are shown in Fig. 5(f) and (g), respectively. Anatomical features such as the optic tectum and the torus longitudinalis can be compared to the H&E-stained histology section, see Fig. 5(h). Furthermore, the pigments found in the fish skin can be identified as highly scattering regions in Fig. 5(f) and (g), respectively.

**Fig. 6.**
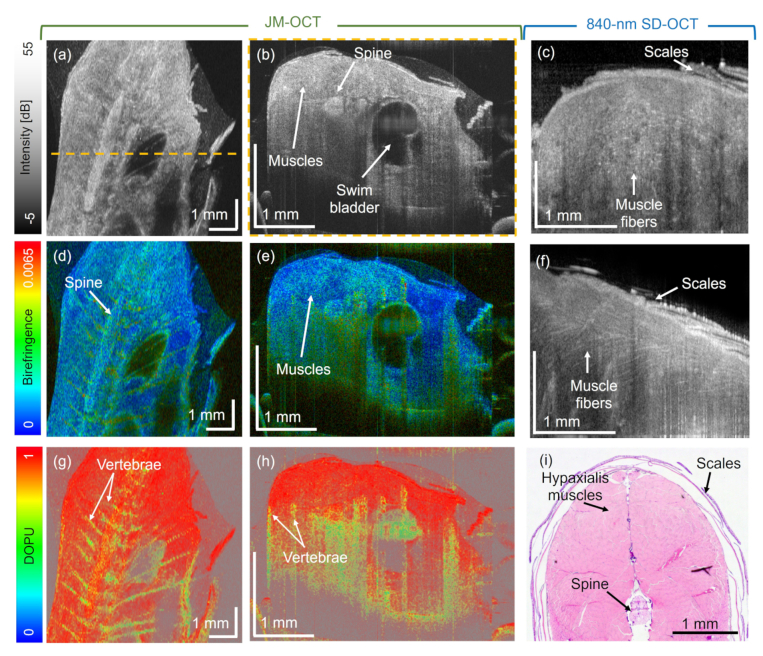
Dorsal muscle region imaged with JM-OCT and 840-nm SD-OCT. (a) - (b) Intensity, (d) - (e) birefringence and (g) - (h) DOPU JM-OCT en-face and B-scan images, respectively. (c) and (f) Intensity based en-face and B-scan images acquired with the 840-nm SD-OCT setup. (i) H&E-stained histology micrograph of the muscle region. The yellow dashed line in (a) indicates the B-scan location in (b).

Finally, the region in the back (see Fig. 1, region (A3)) of a wildtype zebrafish was investigated with both OCT systems. The intensity based en-face and B-scan images acquired with the JM-OCT system are shown in Fig. 6(a) and (b), respectively. The spine shows up as region of high scattering, in contrast to the inside of the swim bladder. These structures can be compared to the H&E-stained micrograph shown in Fig. 6(i). The corresponding birefringent en-face projection and B-scan images are shown in Fig. 6(d) and (e), respectively. In the tomogram in Fig. 6(d) higher birefringence values can be found in the spine and the vertebrae, swim bladder wall and the hypaxial muscle region. Figure 6(g) and (h) show the corresponding DOPU images. The vertebrae and the spine exhibited low DOPU values, see Fig. 6(g). Figure 6(i) shows an H&E-stained transverse section in the investigated region.

The same wildtype fish was imaged using the 840-nm SD-OCT setup. Figure 6(c) shows a tomogram in transverse and (f) in horizontal plane, respectively. A maximum intensity projection of two adjacent B-scans was performed to create these images. Utilizing the 840-nm setup, fine muscle fibers inside the hypaxial muscle region can be visualized as highly scattering structures. The orientation observed of these fibers can be compared to the histology results in Fig. 6(i).

### 3.2 Large field-of-view JM-OCT imaging

**Fig. 7.**
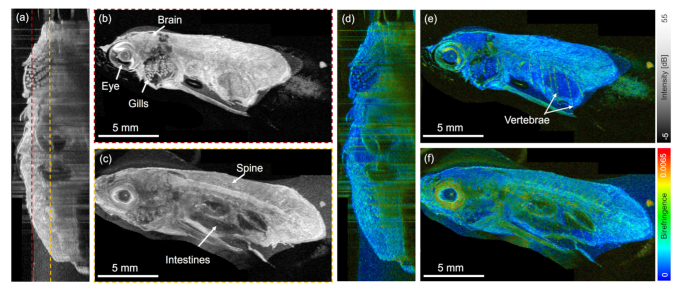
Large FoV imaging with JM-OCT. (a) Sagittal intensity cross section. (b) - (c) En-face images at the positions indicated by the dashed lines in (a). (d) Sagittal birefringence tomogram. (e) - (f) En-face birefringence images.

Utilizing the JM-OCT and the motorized translation stage large FoV images, utilizing three consecutive volumes, were acquired covering the body of the adult zebrafish starting from the head until the middle of the tail. Figure 7 shows the intensity (a) - (c) and birefringence (d) - (f) results, respectively.

A sagittal intensity cross-section through the adult zebrafish body is shown in Fig. 7(a) and selected en-face planes are shown in Fig. 7(b) and (c), respectively. The corresponding birefringence sagittal cross-section and en-face images are shown in Fig. 7(d) - (f), respectively. In the large FoV images anatomical structures such as the eye, the gills, the vertebrae, and muscles can be observed. Utilizing the JM-OCT setup and an imaging range of 6 mm × 6 mm such a large FoV can be acquired in less than a minute.

### 3.3 Investigating tumor zebrafish models with JM-OCT

For this proof-of-concept study, three zebrafish with tumors close to the eye and three with brain tumors in comparison to the equal number of wildtype animals (N=3) were investigated with the JM-OCT prototype. Notice that one fish investigated showed tumor growth near the eye as well as in the brain.

**Fig. 8.**
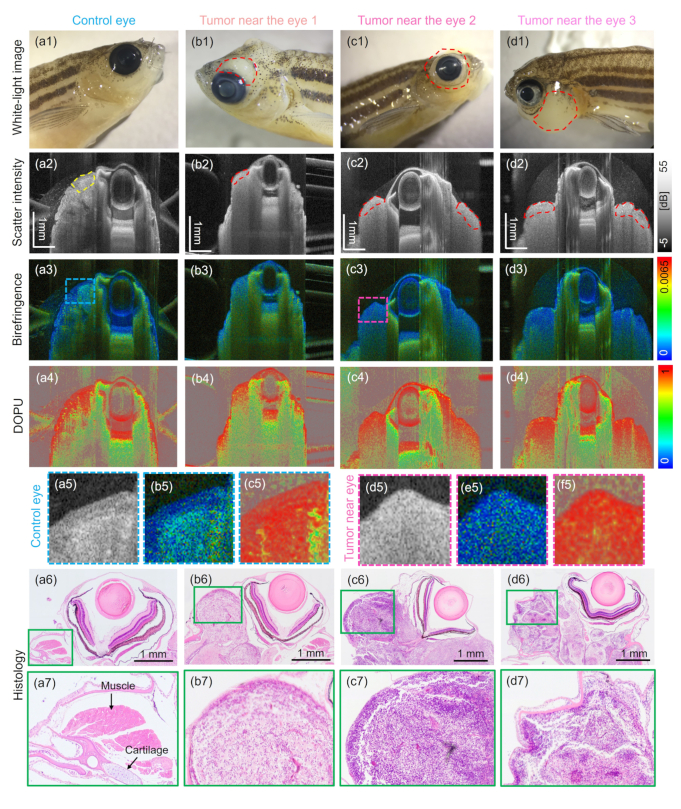
Imaging results obtained in zebrafish models showing tumors near the eye and in one wildtype fish ((a1) - (a6)). (a1) - (d1) White-light photographs of the investigated animals. (a2) - (d2) OCT intensity tomograms. (a3) - (d3) Corresponding birefringence images. (a4) - (d4) Corresponding DOPU images. (a5) - (c5) Zoom-in intensity, birefringence and DOPU images of representative control case. The chosen region is indicated in (a3). (d5) - (f5) Zoom-in intensity, birefringence and DOPU images of representative tumor case. The chosen region is indicated in (c3). (a6) - (d6) H&E-stained histology micrographs with zoom-in images shown in (a7) - (d7), respectively.

Figure 8 gives an overview over all zebrafish models imaged which exhibited tumors close to the eye. The results of a wildtype fish are shown in Fig. 8(a2) - (a6). White-light stereomicroscope images of all zebrafish models are shown in Fig. 8(a1) - (d1), respectively. The tumor affected areas close to the eye are indicated by red dashed lines. The intensity-based tomograms are shown in Fig. 8(a2) - (d2), respectively. Figure 8(a3) - (d3) show the corresponding birefringence and Fig. 8(a4) - (d4) the DOPU images. Additionally, zoom-in images into the intensity, birefringence and DOPU images, respectively for a representative control [Fig. 8(a5) - (c5)] and tumor [Fig. 8(d5) - (f5)] zebrafish are presented. Furthermore, H&E-stained histology micrographs are shown in Fig. 8(a6) - (d6) with additional zoom-in images in Fig. 8(a7) - (d7), respectively. The histology analysis confirmed that the muscle and cartilage areas around the eye were replaced by the tumor.

**Fig. 9.**
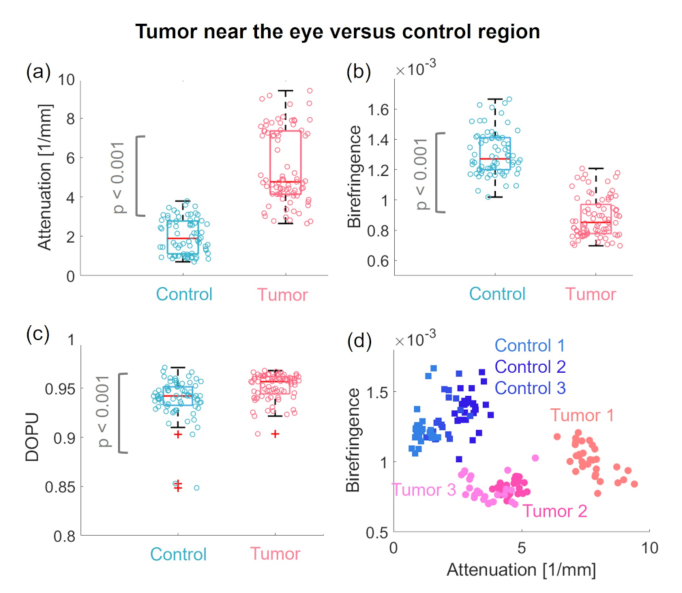
Evaluation of scattering and polarization characteristics in models with tumors near to the eye. (a) Box plot of the attenuation coefficients. (b) Box plot of the birefringence and (c) the DOPU measurements in the tumor in comparison to the control regions. (d) Scatter plot of birefringence values over attenuation coefficients. Control 1 - 3 indicate the measured wildtype fish and Tumor 1 - 3 the three tumor models.

To quantify differences in the scattering and polarization properties in tumor versus control areas, the according values were extracted B-scan-wise by manually segmenting regions of interest as referring to the histology results. The regions to evaluate the scatter and polarization parameters are indicated by yellow and red dashed lines in Fig. 8(a2) – (d2). Box plots of the respective data are shown in Fig. 9(a) - (c), respectively. Each data point represents the mean value of the respective quantity for the annotated region of interest in one B-scan. Please note that an additional random offset in the horizontal axis was added to be able to differentiate individual data points. The results showed significantly higher attenuation coefficients (p < 0.001) in the tumor close to the eye compared to the control region. Further, a significantly lower birefringence (p < 0.001) and significantly higher DOPU (p < 0.001) values were found in the cancer affected regions. Additionally, the Levene’s test revealed a statistically significant difference in the variances for the attenuation data in tumor versus control animals (p < 0.05). Note that the statistical evaluation needs to be interpreted carefully due to the low number of animals and the large heterogeneity of the tumors involved. The scatter plot in Fig. 9(d) shows the birefringence values plotted over the attenuation coefficients for all three cases.

**Fig. 10.**
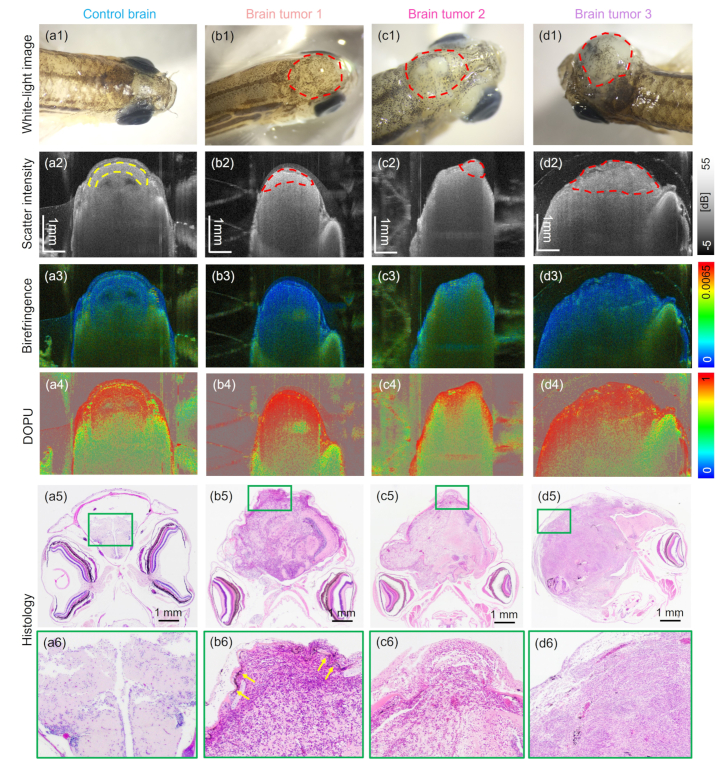
Brain tumor models and one control fish ((a1) - (a6)) imaged with the Jones-matrix optical coherence tomography prototype. (a1) - (d1) White-light photographs of the investigated animals. (a2) - (d2) OCT Intensity-based tomograms. (a3) - (d3) Corresponding birefringence images. (a4) - (d4) Corresponding DOPU images. (a5) - (d5) H&E-stained histology micrographs with zoom-in images shown in (a6) - (d6), respectively. Melanocytes are indicated by yellow arrows in (b6).

Furthermore, brain tumor models were investigated using the JM-OCT prototype, see Fig. 10. The results for a representative control fish are shown in Fig. 10(a2) - (a6). Notice that parts of the optic tectum got detached and that the faint appearance of the control brain is a staining artifact from histology workup. White-light stereomicroscope images of all zebrafish models are shown in Fig. 10(a1) - (d1), respectively. The tumor regions are indicated by red dashed lines. The intensity-based tomograms are shown in Fig. 10(a2) - (d2), respectively. Figure 10(a3) - (d3) show the corresponding birefringence and Fig. 10(a4) - (d4) the DOPU images. Furthermore, the corresponding H&E-stained micrographs are shown in Fig. 10(a5) - (d5) with additional zoom-in images in Fig. 10(a6) - (d6), respectively.

**Fig. 11.**
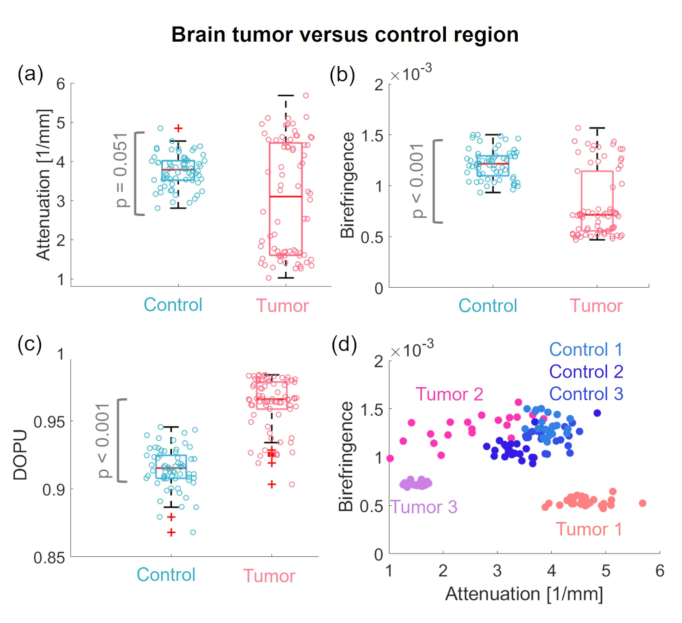
Evaluation of scattering and polarization characteristics in brain tumor models. (a) Box plot of the attenuation coefficients. (b) Box plot of the birefringence and (c) the DOPU values in the brain tumor in comparison to those in the control regions. (d) Scatter plot of birefringence values over attenuation coefficients. Control 1 - 3 indicate the measured control fish and Tumor 1 - 3 the three tumor models.

To quantify differences in the tumor versus control regions in the brain, the measured scattering and polarization characteristics were evaluated. The regions to evaluate the scatter and polarization parameters are indicated by yellow and red dashed lines in Fig. 10(a2) – (d2) and were chosen according to the gained histology results. Box plots of the respective data are shown in Fig. 11(a) - (c), respectively. A decrease in the attenuation coefficients in the tumor compared to the control region was found, however the results were not statistically significant (p value = 0.051). Further, a significant decrease in birefringence (p < 0.001) with a significant increase in DOPU (p < 0.001) values was found. Additionally, the Levene’s test revealed a statistically significant difference in the variances for the attenuation and the birefringence data in tumor versus control animals (p < 0.05). Note that the statistical evaluation needs to be interpreted carefully due to the low number of animals and the large heterogeneity of the tumors involved. The scatter plot in Fig. 11(d) shows the birefringence values plotted over the attenuation coefficients for all animals examined.

## 4. Discussion

The zebrafish is an essential pre-clinical animal model, especially in the field of cancer research [1,2]. For zebrafish-based investigations an imaging modality which can provide a non-destructive, real-time, high-resolution, and three-dimensional way to analyze the anatomical features of these animals is needed. OCT fulfills these requirement and furthermore, PS-OCT can add tissue specific contrast to conventional intensity-based OCT imaging by exploiting the polarization properties of the sample [32].

### 4.1 Adult zebrafish imaging using OCT

#### 4.1.1 Wildtype zebrafish

Utilizing a polarization sensitive JM-OCT prototype with a central wavelength of 1310 nm and a SD-OCT system at 840 nm, adult zebrafish models were investigated. The JM-OCT prototype enabled the examination of tissue specific contrast by analyzing the polarization properties of the anatomical structures in the zebrafish.

First the eye of the zebrafish was imaged, see Fig. 2. The results revealed that the zebrafish eye has similar polarization characteristics as found in humans [32]. The RPE and the choroid showed lower DOPU values (green to yellow) in comparison to the retina, which was polarization preserving. The iris introduced polarization scrambling, which can also be found in murine and human eyes [32,64]. This strong depolarization structures introduced shadowing artefacts, which can be observed as increased birefringent and DOPU trails under the iris and RPE [see Fig. 2(c) - (d)].

Furthermore, utilizing a higher-resolution scanning lens, lens fiber cells [63] were identified with the JM-OCT prototype, see Fig. 2(f) - (g), respectively. These well orientated structures, arranged in concentric shells [65], exhibited higher scattering and birefringence compared to the surrounding connective tissue. In Fig. 2(f) ring structures can be observed, which arise from the orientation of the fiber cells inside the zebrafish lens, see Fig. 3(e). Previous work via histological examination showed that lens epithelium covers the anterior half of the lens fiber core and ends at the equator. At this boundary lens epithelial cells are connected to the lens fiber cells [63], see Fig. 3(e). For the future, it might be interesting to also analyze the axis orientation of these structures [32,64].

The JM-OCT prototype was used to image additional anatomical locations. When analyzing the region of the gills, higher birefringence was found in the cartilage rich regions of the operculum, a multilayered plate protecting the sensitive gills and the surrounding muscles, see Fig. 4(h).

In the dorsal region of the zebrafish, the spinal cord and the vertebrae could be identified by their increased birefringence and reduced DOPU values, see Fig. 6 and Fig. 7, respectively. When comparing these results to literature, an increase in birefringence in myelinated tissue, such as nerve fibers [66,67] as well as in cartilage structures [68,69] was described. The depolarization observed in the vertebrae, [see Fig. 6(g) and (h)] may be caused by the non-spherical, spindle-shaped osteoblast-like cells present in the compact zebrafish bone [70].

Depolarization and therefore decreased DOPU values can be introduced by three main factors, multiple scattering, which can for example be observed in deeper tissue regions, see Fig. 4(b), scattering on non-spherical particles, such as melanin, see Fig. 2(d), or through surface roughness [71]. In anisotropic tissue types, such as in highly orientated muscle fibers, see Fig. 6, an increased birefringence with a high polarization uniformity can be observed. Results found in literature reported similar findings in zebrafish and murine muscles [40,51,66]. Also, the swim bladder wall exhibited high birefringence, see Fig. 6(e), potentially due to the fibrous composition of the supporting musculature layers [72].

#### 4.1.2 Zebrafish tumor model

The data acquired with the JM-OCT setup were utilized to investigate scattering and polarization changes in wildtype fish versus models showing tumors near the eye and in the brain. In the models exhibiting tumors near the eye, the histological analysis confirmed that in all three cases the tumor mass replaced the adjacent muscle and cartilage structures, see Fig. 8. Two out of three tumor cases showed increased attenuation coefficients in comparison to the control regions [see Fig. 9(d)], which might be caused by the stronger backscattering of the densely packed tumor mass in comparison to the low-scattering signature of healthy muscle tissue. Further, decrease in birefringence in the tumor compared to the control area was measured [see Fig. 9(b)], which can again be traced back to the replaced muscle tissue by the disorganized tumor mass, see Fig. 8. Please note that these results need to be interpreted with care, due to the small sample size and the heterogeneous manifestation of these tumors.

The brain showed like the tumors near to the eye an intra-tumor heterogeneity. Though, in all cases a disruption of the healthy brain morphology in comparison to the control region can be observed, see Fig. 10. Decrease in attenuation values for two out of the three tumor cases versus the control areas was found, see Fig. 11(d). Decreased attenuation coefficients in some types of brain tumors have been reported in murine models and humans [73,74]. The histology analysis, Fig. 10, revealed that the tumor areas showed an increased density of malignant cells. Literature has suggested that this can ultimately lead to decreased attenuation values in specific brain tumor types [73].

However, one brain tumor case (brain tumor 1) exhibited similar attenuation values compared to the control brains. Histology analysis revealed that this tumor had a large amount of melanocytes present [indicated by yellow arrows in Fig. 10(b6)] which might explain the elevated scattering behaviour. When analyzing the polarization parameters, a significant decrease in birefringence with a significant increase in DOPU values in tumor versus control region was measured, see Fig. 11(b) and (c), respectively. A decrease in birefringence has been described in literature for tumors found in human white matter due to the loss of myelin [75]. Though, one case (brain tumor 2) showed a similar birefringence signature as the control brains. The corresponding histology image [Fig. 10(c5) - (c6)] revealed that this case in contrast to the others showed a lower nuclei density and the presents of more white matter.

Axis orientation has shown to be a great tool to identify the structure of white matter in brain tissue [67]. Our JM-OCT is a single-mode-fiber based system, and it is not straight forward to measure absolute optic axis orientation. However, there are several recent studies measuring axis orientation by using single-mode-fiber based PS-OCT [76,77]. It encourages us to implement a depth-resolved axis orientation analysis as an additional polarization-sensitive parameter to improve the identification of abnormalities.

By analyzing the scattering, the birefringence and depolarization information gained by the JM-OCT setup different tissue features can be investigated. The scatter-based information provided the general morphology of the tissue. In addition, the birefringence is sensitive to tissue containing highly orientated structures, such as muscle, nerve fibers, or collagen [Fig. 4(b) and (h) and Fig. Fig. 6(d) and (e)]. In the diseased model showing tumor growth next to the eye [Fig. 8], this contrast channel revealed the alteration of the adjacent healthy muscle region. In the brain tumor model, [Fig. Fig. 10] the birefringence detected the disruption of the white matter content by the tumor mass. DOPU is a marker for multiple-scattering or scattering on non-spherical particles. As such it identifies highly pigmented structure such as the sclera or RPE [Fig. 2(d)] and cartilage rich areas [Fig. Fig. 4(c) and (i) and Fig. 6(g) and (h)]. The increased DOPU and therefore decreased depolarization characteristic found in brain tumors and tumors next to the eye, might be explained by the homogeneity of the neoplasm structures investigated.

#### 4.1.3 Future steps

For both tumor models an intra-tumor variability was observed, see Fig. 9(d) and 11(d). One limitation of these analyses was the small sample size, in the future, more animals needs to be analyzed to get stronger statistical results and gain a deeper knowledge about scattering and polarization changes inside the tumor types. An additional feature based structural analysis of the acquired data could further support the identification of abnormalities [78].

### 4.2 Effect of multiple scattering

The theoretical depth range in air of the JM-OCT setup was 2.9 mm. However due to multiple factors, like attenuation, multiple scattering, and the depth of focus of the used scanning lenses around 1.5 mm in depth could be examined with a high signal to noise ratio.

High birefringence and low DOPU values were observed in the bottom region of most images (see Fig. 4(b) and (c) or Fig. 6(e) and (h)). This artefact may arise from multiple scattering at deeper tissue regions [71]. In the future, methods combining hardware improvements and signal processing could suppress this artefact and may lead to an increased imaging depth [79–82].

Additionally, high depolarization, for example introduced by the densely pigmented fish iris (see Fig. 2(c) and (d)) can also lead to increased birefringence values. The interpretation of birefringence values at low DOPU regions is still an open issue and JM-OCT images need to be interpreted with care. In the future, we plan to classify tissue structures by utilizing a machine learning algorithm for both polarization properties simultaneously, where the birefringence values at regions of high DOPU can safely be quantified.

### 4.3 Comparison of 1310-nm and 840-nm imaging

When comparing the results obtained with 840 nm and 1310 nm differences were observed. Due to the shorter central wavelength and the larger bandwidth of the used light source, the axial resolution of the 840-nm SD-OCT setup was around three times higher than in the JM-OCT system, i.e. 5.3 μ m for SD-OCT and 14 μ m for JM-OCT. Further, stronger backscattering at various tissue structures can be observed at shorter wavelengths [83]. Due to the lower central wavelength, also the lateral resolution was higher even though a similar scanning lens was utilized. All these factors enabled, for example, the visualization of the fiber cells throughout the whole lens (see Fig. 3) or the fine details in the branches of the gills (see Fig. 5). For the future it might be interesting to develop a JM-OCT setup which utilizes a light source in lower wavelength ranges to further investigate the polarization properties of various zebrafish organs.

However, by utilizing the 1310-nm JM-OCT system, a deeper imaging depth was achieved and therefore a big portion of the adult zebrafish body can be visualized, see Fig. 7. For the 840-nm setup sectioning and stitching of multiple volumetric acquisitions would be needed to generate similar depth ranges [67,84]. The utilized 1310-nm JM-OCT prototype showed to be a promising tool for non-destructive and high-resolution anatomical feature examination in adult zebrafish models.

Further, a potential problem of the current JM-OCT implementation is that the depth range is limited due to the depth multiplexing of two incident polarizations. This becomes problematic when implementing Jones matrix SD-OCT, which possibly has high resolution as we demonstrated with 840-nm SD-OCT in this manuscript, but has a more limited depth range than SS-OCT. One possible solution is using a swept source laser in the 840-nm wavelength region [85].

Please note that the results presented for the 1310-nm and 840-nm setups were not co-registered. In next measurements, a bio-compatible marker could be applied, to be able to image the exact same location to overcome this limitation.

### 4.4 Large field-of-view imaging

An automated x - y translation stage in combination with the JM-OCT prototype enabled large FoV imaging and nearly the whole adult zebrafish body could be examined in less than a minute, see Fig. 7. In the current study, the locations of the tumor regions have been known from histology before evaluating the OCT images. One of the final goals of the zebrafish investigation is a fast and non-destructive screening and phenotyping of cancer models, however, in which the exact anatomical manifestations of the tumor are unknown. We are planning to identify scatter and polarization-based threshold values in combination with deep learning methods to facilitate an automated identification of abnormal regions. Furthermore, the combination of the scatter and polarization information can be used to generate pseudo color maps for a more comprehensive visualization of the data.

### 4.5 Advantages of OCT and outlook

OCT in comparison to the gold-standard of histology, enabled real-time and label-free imaging of the adult zebrafish. Another advantage of the presented approach is that it is non-invasive as it is based on low-coherence interferometry of light. Namely, no harmful radiation or high magnetic fields such as for MRI or CT are needed [9,18].

Already during the imaging session, a real-time examination of the tissue features based on the scattering information can be performed. In the future using GPU based-processing a life preview of the polarization characteristics of the zebrafish anatomy could further be implemented.

The JM-OCT prototype was running at an A-scan rate of 50 kHz which enables real-time imaging [86]. Therefore, as a next step we would like to focus on *in vivo* tumor zebrafish model investigations. When performing *in vivo* measurements dynamic processes like the blood flow utilizing OCT angiography (OCTA) [87] or even more subtle intracellular changes using dynamic OCT (D-OCT) [88] can be investigated. Recently, we developed a D-OCT imaging protocol which allowed us to identify the viability of tissue in a micrometer scale [28]. For the future this algorithm will be applied to *in vivo* data to identify anatomical features and abnormalities with an additional contrast channel. Furthermore, the importance of OCTA in various animal models in observing changes in the vasculature as a biomarker for tumor growth has been shown [89]. Taking all this into account we are planning a longitudinal, non-invasive study to investigate tumor growth in a zebrafish model utilizing D-OCT and OCTA in combination with our JM-OCT prototype.

## 5. Conclusion

In conclusion, in this article we presented the investigation of adult zebrafish models utilizing two OCT systems, a polarization sensitive JM-OCT prototype at 1310 nm and a high-resolution SD-OCT system with a central wavelength of 840 nm. Anatomical features of the zebrafish such as the eye, the gills, the brain, and the muscles were examined with high-resolution and in a non-destructive way. The polarization and scattering characteristics of these structures were identified. Furthermore, a motorized translation stage enabled the investigation of large areas in the adult zebrafish in combination with the JM-OCT prototype. Finally, zebrafish tumor models were examined. The scattering and polarization properties found in the tumor in comparison to control regions were analyzed. Tumors near the eye and in the brain showed a significant decrease in birefringence compared to control regions; however, one limitation of this study was the small sample size. In the future, a higher number of animals needs to be analyzed to get stronger statistical results and gain a deeper knowledge about scattering and polarization changes inside the tumor versus the control tissue. The presented work showed the potential of OCT and JM-OCT as non-invasive, high-resolution, and real-time imaging tools in the field of pre-clinical zebrafish-based research.

## Data Availability

Data underlying the results presented in this paper are not publicly available at this time but may be obtained from the authors upon reasonable request.
